# Where to Publish: helping health sciences professionals find journals for publication quickly and safely

**DOI:** 10.5195/jmla.2021.1355

**Published:** 2021-10-01

**Authors:** Matt Weaver

**Affiliations:** 1 weaverm2@ccf.org, Systems Medical Librarian, Cleveland Clinic Floyd D. Loop Alumni Library, Cleveland, OH

## BACKGROUND

Academic writing is something that health sciences professionals work into their busy schedules. With the rise of open access publishing, the environment has become harder for authors to navigate.

Authors have several key concerns when seeking publication: finding journals likely to accept the paper, identifying open access journals, and avoiding so-called predatory journals. Often, they turn to the library for answers.

Separately, each concern can be time-consuming and difficult to address. The library wanted to create a user-friendly, reliable, and expedient website to handle these requests, saving the time of both our patrons and library staff.

In May 2019, the Cleveland Clinic Floyd D. Loop Alumni Library launched Where to Publish, a website that takes a user's keyword search and returns data to address all of these concerns. Due to content restrictions from Clarivate Analytics, Where to Publish is only available on the Cleveland Clinic local network.

## METHODS

Where to Publish was built in the Python programming language. The library chose the Flask web framework because it is lightweight, well supported, and appropriate for small websites.

WTForms, a Python module for generating and validating form input in web platforms, receives the user's search terms. The site's core script uses the Biopython module, which includes the National Library of Medicine's (NLM) Entrez application programming interface (API), to return journals ranked by the number of times the journal has published papers that fit the search criteria.

Where to Publish uses Clarivate Analytics' journal impact factor (JIF) as an indicator of journal quality in two respects. First, JIF is used by Cleveland Clinic in physicians' annual performance reviews. Second, by providing a list of journals with JIFs, Where to Publish ensures that authors will avoid low-quality journals due to Clarivate's rigorous journal selection criteria [1], especially as predatory journals have been getting their papers into PubMed via PubMed Central [2].

Sorting by JIF works as follows:

- A file is created using the NLM's eDirect command line utility that contains all NLM catalog journal records and data fields like ISSNs.
- For each search, the core Python script passes PubMed IDs to the Web of Science APIs to retrieve papers-level metadata from Web of Science.
- The script compares the papers' ISSNs against the journal list generated in eDirect and produces tables of journals with and without JIFs.
- Open access indicators and additional metrics are attached for journals with JIFs.

## RESULTS

In its first full year, May 2019 to May 2020, Where to Publish was searched 413 times. Usage was affected by the COVID-19 pandemic as Cleveland Clinic Lerner College of Medicine students and many residents were sent home in March 2020. Removing similar searches by users as they refined results, the number of unique search topics was 206. If all unique search topics became papers, they would represent around 3.4% of the some 6,000 papers a year published by Cleveland Clinic in Web of Science–indexed journals [3].

## DISCUSSION

Where to Publish is being used by Cleveland Clinic authors to locate relevant journals for publication.

Future development includes a revision of the data in the output so it is easier to read and the addition of journals' URLs so users can get to submission guidelines quickly.

In addition, the library is working on a separate, bespoke search for authors in nursing and allied health professionals. This interface will use inclusion in CINAHL as a quality indicator. PubMed is no longer supporting its Nursing Journals filter [4], so using CINAHL's API will ensure that authors receive a reliable list of relevant journals.

**Figure 1:**
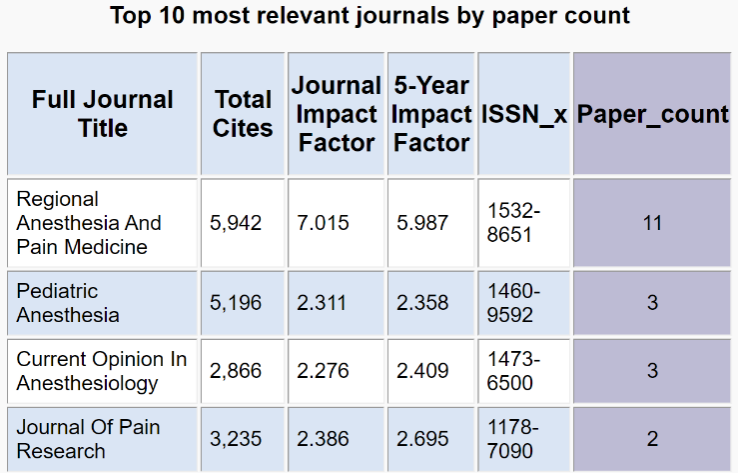
Journals ranked by the number of papers published on the search topic. Sample keyword search: chest wall blocks.

**Figure 2:**
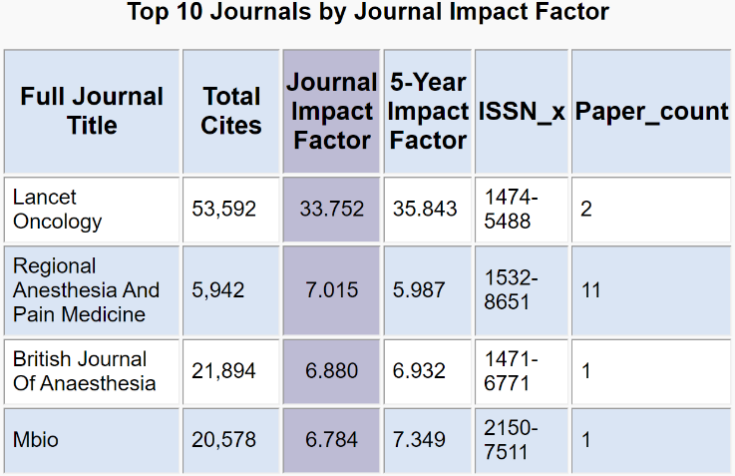
Journals ranked by their JIF. Sample keyword search: chest wall blocks.

**Table 1:** Open access journals. Sample keyword search: chest wall blocks.

Open access journals
*Journal of Pain Research*
*BMC Medical Imaging*
*Anatolian Journal of Cardiology*
*PLOS One*
*Pain Research & Management*
*mBio*
*The Korean Journal of Pain*
*Scientific Reports*
*BioMed Research International*
